# Identification of a Novel Lipidomic Biomarker for Hepatocyte Carcinoma Diagnosis: Advanced Boosting Machine Learning Techniques Integrated with Explainable Artificial Intelligence

**DOI:** 10.3390/metabo15110716

**Published:** 2025-11-01

**Authors:** Fatma Hilal Yagin, Cemil Colak, Fahaid Al-Hashem, Sarah A. Alzakari, Amel Ali Alhussan, Mohammadreza Aghaei

**Affiliations:** 1 Department of Biostatistics, Faculty of Medicine, Malatya Turgut Ozal University, Malatya 44210, Türkiye; 2 Department of Computer Science, Lakehead University, Thunder Bay, ON P7B 5E1, Canada; 3 Department of Biostatistics, and Medical Informatics, Faculty of Medicine, Inonu University, Malatya 44280, Türkiye; 4 Department of Physiology, College of Medicine, King Khalid University, Abha 61421, Saudi Arabia; 5 Department of Computer Sciences, College of Computer and Information Sciences, Princess Nourah Bint Abdulrahman University, Riyadh 11671, Saudi Arabia; 6 Department of Ocean Operations and Civil Engineering, Norwegian University of Science and Technology (NTNU), 7034 Alesund, Norway

**Keywords:** hepatocellular carcinoma, lipidomics, biomarkers, machine learning, explainable boosting machine

## Abstract

**Background:** Hepatocellular carcinoma (HCC) is a leading cause of cancer-related mortality worldwide, often diagnosed at late stages due to the limited sensitivity of current screening tools. This study explores whether blood-based lipidomic profiling, combined with explainable artificial intelligence (XAI), can improve early and interpretable detection of HCC. **Methods:** We analyzed lipidomic data from 219 HCC patients and 219 matched healthy controls using liquid chromatography-mass spectrometry. An Explainable Boosting Machine (EBM) was employed to identify discriminatory lipid biomarkers and was compared against several standard machine learning algorithms. **Results:** The EBM model achieved superior performance with 87.0% accuracy, 87.7% sensitivity, 86.3% specificity, and an AUC of 91.8%, outperforming other models. Key lipid biomarkers identified included specific phosphatidylcholines (PC 38:2, PC 40:4), sphingomyelins (SM d40:2 B), and lysophosphatidylcholines (LPC 18:2), which exhibited significant alterations in HCC patients and highlighted disruptions in sphingolipid metabolism. **Conclusions:** Integration of lipidomics with explainable machine learning offers a powerful, transparent approach for HCC biomarker discovery, achieving high diagnostic accuracy while providing biological insights. This strategy holds promise for developing non-invasive, clinically interpretable screening tools to improve early detection of liver cancer.

## 1. Introduction

Hepatocellular carcinoma (HCC) represents one of the most prevalent and lethal forms of primary liver cancer worldwide, accounting for approximately 90% of all liver malignancies. As the sixth most common cancer and the third leading cause of cancer-related deaths globally, HCC imposes a substantial burden on public health systems, with over 900,000 new cases and 830,000 deaths reported in 2020. Projections indicate that by 2040, the incidence and mortality rates of HCC could rise by more than 55%, driven by factors such as increasing prevalence of metabolic disorders, viral hepatitis, and lifestyle-related risks. In regions like East Asia and sub-Saharan Africa, chronic hepatitis B virus (HBV) infection remains a dominant etiological factor, while in Western countries, non-alcoholic fatty liver disease (NAFLD) and metabolic syndrome are emerging as primary contributors, reflecting a shifting epidemiological landscape. The United States, for instance, has seen HCC incidence rates triple since 1980, stabilizing only recently, yet mortality continues to climb, underscoring the urgent need for improved early detection and management strategies [1,2,3,4].

The prognosis for HCC remains poor, with a five-year survival rate often below 20%, largely due to late-stage diagnosis and limited therapeutic options. Early-stage HCC is frequently asymptomatic, leading to detection at advanced stages where curative interventions like surgical resection or liver transplantation are no longer viable. Current diagnostic tools, such as serum alpha-fetoprotein (AFP) levels and imaging modalities including ultrasound, computed tomography (CT), and magnetic resonance imaging (MRI), suffer from suboptimal sensitivity and specificity, particularly in distinguishing HCC from benign liver conditions like cirrhosis. For example, AFP has a sensitivity of only 40–65% for early-stage HCC, often resulting in false negatives or positives that complicate clinical decision-making. Moreover, risk stratification and prognostic assessments rely heavily on clinical staging systems like the Barcelona Clinic Liver Cancer (BCLC) criteria, which, while useful, do not fully capture the molecular heterogeneity of HCC. These limitations highlight the necessity for novel biomarkers that can enable non-invasive, accurate early detection and personalized risk assessment [5,6,7].

Recent advances in omics technologies have illuminated the molecular underpinnings of HCC, revealing profound metabolic reprogramming as a hallmark of the disease. Among these, lipidomics—the comprehensive study of lipid species and their interactions—has emerged as a promising avenue for uncovering dysregulated pathways in HCC). Lipids, including phospholipids, sphingolipids, and fatty acids, play pivotal roles in cellular membrane integrity, signaling, and energy homeostasis, and their alterations in HCC reflect the tumor’s adaptation to hypoxic and nutrient-deprived environments. Studies have demonstrated that HCC tissues exhibit elevated levels of saturated triacylglycerols (TAGs) and reduced polyunsaturated fatty acids (PUFAs), correlating with disease severity and progression. The aberrant accumulation of ceramides and sphingomyelins disrupts apoptosis and promotes inflammation, contributing to fibrosis and carcinogenesis. These lipid perturbations not only serve as potential biomarkers but also align with the rising incidence of NAFLD-associated HCC, where hepatic steatosis precedes malignant transformation [8,9,10,11,12].

The integration of lipidomics with machine learning (ML) algorithms represents a transformative approach to harnessing these metabolic insights for clinical utility. ML excels in processing high-dimensional, complex datasets [13,14] like those from lipidomic profiling, enabling pattern recognition, feature selection, and predictive modeling that surpass traditional statistical methods. Furthermore, ML facilitates multi-omics integration, combining lipidomics with genomics and proteomics to uncover synergistic biomarkers, as demonstrated in prognostic models for HCC survival [15,16]. However, the “black-box” nature of many ML models poses a significant barrier to their adoption in healthcare, where interpretability is paramount for building trust and ensuring ethical decision-making. Explainable artificial intelligence (XAI) addresses this by providing mechanisms to elucidate model predictions, such as feature importance scores or visual saliency maps, allowing clinicians to understand the rationale behind AI outputs [17]. In medical applications, XAI enhances accountability, reduces bias, and complies with regulatory frameworks like the European Association for the Study of the Liver (EASL) guidelines, which emphasize evidence-based practices. XAI techniques like SHapley Additive exPlanations (SHAP) have been used to interpret lipid-based ML models in HCC, revealing how specific ceramides influence prognostic scores. This transparency is crucial in high-stakes scenarios, where erroneous predictions could lead to delayed interventions or overtreatment [18,19,20,21].

The convergence of lipidomics, ML, and XAI holds immense promise for precision medicine in HCC. By identifying lipid biomarkers through advanced analytics, these tools can enable non-invasive screening in high-risk populations, such as those with chronic HBV or NAFLD, potentially reducing mortality by facilitating earlier therapeutic interventions. Moreover, explainable models can guide mechanistic studies, elucidating how sphingolipids modulate immune responses or how ceramides drive fibrosis, paving the way for targeted therapies. Despite challenges like data heterogeneity and model generalizability, ongoing developments in federated learning and multi-center validations are addressing these gaps [11,12,22].

This study leverages lipidomic data from HCC patients and matched controls to develop an explainable boosting machine (EBM) model for classification and biomarker discovery. To address these goals, we performed a secondary computational analysis of publicly available lipidomics data from the Metabolomics Workbench (Study ID: ST002764), initially generated in a nested case–control study in the ATBC cohort. By integrating untargeted lipid profiling with advanced ML and XAI techniques, we aim to identify key lipid alterations, evaluate model performance against traditional algorithms, and provide interpretable insights into HCC pathogenesis. Our findings underscore the potential of lipid-based XAI frameworks to enhance diagnostic accuracy, prognostic stratification, and therapeutic innovation in HCC, ultimately advancing patient-centered care.

## 2. Materials and Methods

### 2.1. Participants and Lipidomic Data

This study represents a computational re-analysis of publicly accessible lipidomic data retrieved from the Metabolomics Workbench (https://www.metabolomicsworkbench.org accessed on 12 August 2025, Study ID: ST002764). The dataset originated from a case–control study comprising 219 individuals diagnosed with liver cancer and an equal number of matched controls. Blood specimens were collected and analyzed through high-resolution, untargeted lipidomic profiling based on mass spectrometry. Individuals with any malignancy other than non-melanoma skin cancer, advanced-stage cirrhosis, chronic alcohol use, or medical conditions that could impede participation were excluded. Control participants were matched to cases in a 1:1 ratio considering age at randomization (±5 years), date of sample collection (±30 days), number of freeze–thaw cycles, and the laboratory responsible for prior aliquoting. Complex lipids were quantified semi-quantitatively from blood samples using liquid chromatography coupled with quadrupole time-of-flight mass spectrometry (LC-QTOF-MS) in an untargeted analysis approach [23]. It should be noted that the publicly available dataset utilized in this study did not include detailed clinical characteristics such as HCC etiology, tumor stage, or specific comorbidities. While this limits our ability to perform subgroup analyses, the dataset’s strength lies in its well-matched case–control design and comprehensive lipidomic profiling, which allowed for robust initial biomarker discovery focused on general HCC detection. A dual-polar acquisition approach was used to acquire lipidomic data from the database: positive electrospray ionization mode (ESI+) using an Agilent 6530 QTOF and negative electrospray ionization mode (ESI-) using an Agilent 6550 QTOF. Full-scan data were acquired in the mass range *m*/*z* 65–1700 in both modes. Lipid identification was achieved by accurate mass matching and retention time alignment against reference spectral libraries, as described in the original study [20]. Lipid identification was performed using accurate mass, retention time, adduct pattern, and in silico spectral matching against the LipidBlast library within the standardized workflow of the West Coast Metabolomics Center. Because experimental MS/MS data were not acquired in this platform, all lipids are putatively annotated (MSI Level 2–3) rather than confirmed at Level 1 [24]. Nonetheless, key lipid species highlighted by the EBM analysis, such as PC(38:2) and SM(d40:2 B), were among those with highly consistent chromatographic and mass features, supporting the robustness of our findings.

### 2.2. Machine Learning Process and Explainability

Support Vector Machine (SVM), CatBoost, Random Forest, XGBoost, and EBM algorithms were used in the classification of liver cancer. SVM is a classification technique that transforms data into a high-dimensional space and determines the hyperplane that provides the widest margin between classes. CatBoost is a gradient boosting-based ensemble method optimized to prevent overfitting. Random Forest is an ensemble model that combines the results of multiple decision trees created with randomly selected samples and features. XGBoost is an ensemble algorithm that optimizes gradient boosting logic with speed and regularization to achieve high accuracy. EBM is an XAI approach that produces both high accuracy and transparent, interpretable results by modeling the effect of each feature separately [25,26,27]. All data processing was implemented in Python 3.9 using the scikit-learn 1.2.0, XGBoost 1.7.0, CatBoost 1.2, and InterpretML 0.4.0 libraries. The following hyperparameters were used: For SVM, we used a radial basis function (RBF) kernel with C = 1.0 and gamma = ‘scale’. Random Forest used 100 estimators with max_depth = 10 and min_samples_split = 5. XGBoost parameters included learning_rate = 0.1, max_depth = 6, n_estimators = 100, and subsample = 0.8. For CatBoost, we used iterations = 1000, learning_rate = 0.1, and depth = 6. EBM used max_bins = 256, and outer_bags = 8. All models used a random starting point of 42 for repeatability. Identical data partitions were used across models, and each algorithm underwent parameter tuning using grid-based optimization within the same search space constraints. This consistent preprocessing and tuning procedure ensured that performance differences reflected model architecture rather than data handling or optimization bias. To ensure robust performance estimation and reduce variance in evaluation metrics, the dataset was split using a 100-fold holdout validation approach with an 80–20 train-test split (stratified by class labels). Therefore, the training and testing process was repeated 100 times to avoid reporting biased estimation results. Performance comparisons were conducted using accuracy, F1 score, sensitivity, specificity, and AUC metrics. The EBM model provided biological interpretability by detailing the effects of lipids on classification decisions through global and local explanations [28].

### 2.3. Statistical Analysis

Descriptive statistics were calculated for the baseline characteristic. Normality of continuous variables was assessed using the Shapiro–Wilk test. Variables with normal distribution are presented as mean ± standard deviation (SD). Categorical variables are expressed as frequencies with percentages. Lipid measurements represent semi-quantitative integrated peak areas expressed in arbitrary units (a.u.), as is standard practice in untargeted lipidomics workflows. The lipidomics dataset obtained was free of missing values. Batch effects in the corresponding data were mitigated by the use of quality control (QC) samples interspersed throughout the analytical sequence. Data normalization was performed using total ion current (TIC) normalization to account for variations in sample concentration and ionization efficiency, as implemented in the original Metabolomics Workbench dataset [20]. Internal standards (ISTDs) were used for quality control and instrument performance monitoring in the analytical process, but these compounds were excluded from our statistical and ML analyses because they were not biologically relevant targets for intergroup comparisons. The focus of our research is to identify biologically meaningful lipid changes through explainable machine learning, rather than determining absolute quantitative concentrations. This semi-quantitative approach is suitable for biomarker discovery studies, where relative differences between disease and control groups are validated with both traditional statistics and interpretable machine learning models, providing actionable clinical information. For statistical analysis, Probability Quotient Normalization (PQN) was first applied to the lipidomic data. The data were then log_2_ transformed and autoscaled. For univariate analysis, differences in lipid concentrations were assessed for statistical comparison using fold change and a non-parametric t-test. With fold change analysis, the q-value was obtained using the Benjamini–Hochberg correction for the *p*-value. The q-value was used to assess statistical significance between the two analysis groups. Differential metabolites were defined according to the following criteria: fold change ≥ 1.2 and q-value < 0.05. Results are presented in tables and Volcano plots. For multivariate analysis, a Partial Least Squares-Discriminant Analysis (PLS-DA) model was applied using permutations. For model optimization, 5-fold cross-validation was used, exploring a maximum of 5 components. Model performance was evaluated using accuracy, R^2^, and Q^2^ values. Ward’s method and Euclidean distance measure were used for hierarchical cluster analysis. In the heatmap visualization, the top 25 most significant features were examined according to PLS-DA VIP scores using Ward’s method and Euclidean distance measure. Statistical significance was accepted as *p* < 0.05. Relevant analyses were performed using Python 3.9 version the MetaboAnalystR Package 4.0.0.

## 3. Results

Table 1 summarizes the demographic and clinical characteristics of the study population. The mean age was approximately 57 years in both groups, with similar age distributions observed across categories. Notable differences emerged in several risk factors between cases and controls. Body mass index was significantly elevated among cases, particularly with a higher prevalence of obesity (BMI ≥ 30 kg/m^2^: 22.8% vs. 13.2%). Cases demonstrated more intensive smoking patterns, with over one-third reporting ≥ 45 pack-years of exposure compared to approximately one-fifth of controls. Alcohol consumption followed a similar trend, where cases were nearly twice as likely to consume ≥ 2 drinks daily (28.3% vs. 16.4%). The prevalence of diabetes was markedly higher in cases relative to controls (9.6% vs. 2.3%). Educational background showed a trend toward higher attainment among cases (Table 1).

Lipidomic profiling analysis identified 15 lipid species that showed statistically significant differences between the two groups (FC ≥ 1.2, q < 0.05). The analysis revealed increased levels of 8 lipid species and decreased levels of 7 lipid species in HCC patients compared with healthy controls. Among the lipids with up regulation, the highest fold change value was observed in FA 22:2 (docosadienoic acid) (log_2_FC = 0.599, q < 0.001), followed by PC 32:1 (log_2_FC = 0.471, q < 0.001), FA 16:1 (palmitoleic acid) (log_2_FC = 0.431, q < 0.001), Ceramide d40:0 (log_2_FC = 0.348, q < 0.001), PE 34:1 (log_2_FC = 0.333, q < 0.001), PC 40:4 (log_2_FC = 0.314, q < 0.001), PC 40:5 B (log_2_FC = 0.286, q < 0.001), SM d42:0 (log_2_FC = 0.280, q = 0.008) and Ceramide d42:0 (log_2_FC = 0.275, q < 0.001) followed. Among the lipids with down regulation, the most significant change was detected in SM d39:2 (log_2_FC = −0.434, q < 0.001), followed by SM d36:3 (log_2_FC = −0.346, q < 0.001), PC p-42:5 or PC o-42:6 (log_2_FC = −0.297, q = 0.003), SM d40:2 B (log_2_FC = −0.285, q < 0.001), LPC 18:2 (log_2_FC = −0.274, q < 0.001) and SM d37:1 (log_2_FC = −0.272, q < 0.001) (Supplementary Table S1 and Figure S1). According to PLS-DA results, the permutation test revealed that the model was statistically significant and moderately able to distinguish hepatocellular carcinoma patients from controls based on lipidomic profiles, indicating a moderate R^2^ value. Therefore, complex machine learning approaches were applied for more robust classification. Hierarchical clustering analysis and heat mapping revealed that the 25 lipids with the highest VIP scores showed significant expression differences between the patient and control groups, and samples clustered by group (Supplementary Figures S2–S6).

In a performance comparison of machine learning models developed for the classification of liver cancer using lipidomic data, the EBM model showed the most robust performance across all performance metrics. EBM outperformed other models in terms of accuracy with 87.0% (0.838–0.901), while achieving an F1 score of 87.1% (0.839–0.902), sensitivity of 87.7% (0.826–0.917), and specificity of 86.3% (0.810–0.906). In addition, the AUC value of EBM was 0.918 (0.870–0.966), which best reflects its classification power and robustness. In comparison, the XGBoost model achieved the closest performance, with an accuracy of 80.6% (0.769–0.843), an F1 score of 81.2% (0.776–0.849), sensitivity of 84.0% (0.785–0.886), specificity of 77.2% (0.710–0.826), and an AUC of 0.866 (0.806–0.927). Although Random Forest, CatBoost, and SVM models also demonstrated stable performance, they lagged behind EBM and XGBoost models in all performance metrics. These results demonstrate that the EBM model is a strong candidate for clinical decision support systems, exhibiting superior performance in lipidomics-based liver cancer classification in terms of both overall accuracy and balance metric (F1), sensitivity, specificity, and AUC (Table 2).

**Figure 1:**
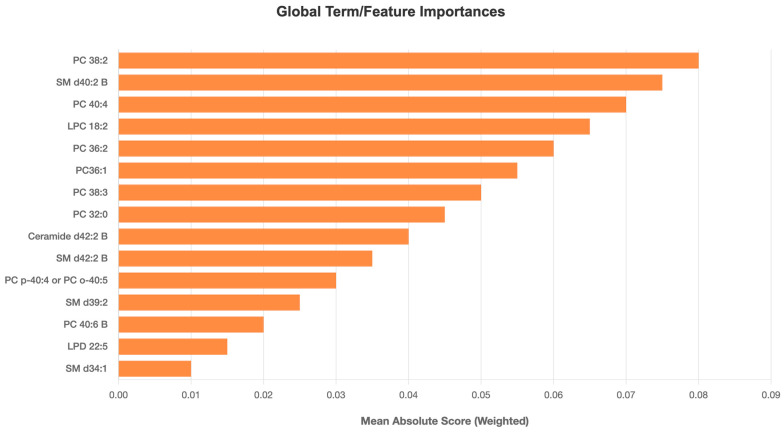
Global explanation of the Explainable Boosting Machine (EBM) model for hepatocellular carcinoma (HCC) classification. The plot shows the average absolute contribution of each lipid feature to the model output, representing its overall importance in distinguishing HCC from healthy controls. Abbreviations: PC—phosphatidylcholine; SM—sphingomyelin; LPC—lysophosphatidylcholine; Cer—ceramide; LPD—Lysophosphatidyl derivatives. The *x*-axis shows lipid features name ranked by importance; the *y*-axis displays average contribution scores to the EBM prediction.

The global explanation obtained for the EBM model’s liver cancer classification using lipids clearly reveals which lipids have the highest weight in the model’s decision-making process. Figure 1 shows that lipid types such as PC (38:2), SM d40:2 B, PC (40:4), LPC (18:2), PC (36:2), and PC (36:1) are ranked highest in terms of average absolute score values. These lipids stand out as the predictors contributing most to the model’s classification performance. Other lipid types such as Ceramide d42:2 B, and SM d42:2 B, also have significantly higher importance scores. These results support the potential of specific saturated, unsaturated, and phospholipid compounds as liver cancer biomarkers and demonstrate the EBM model’s ability to highlight biologically significant features.

**Figure 2:**
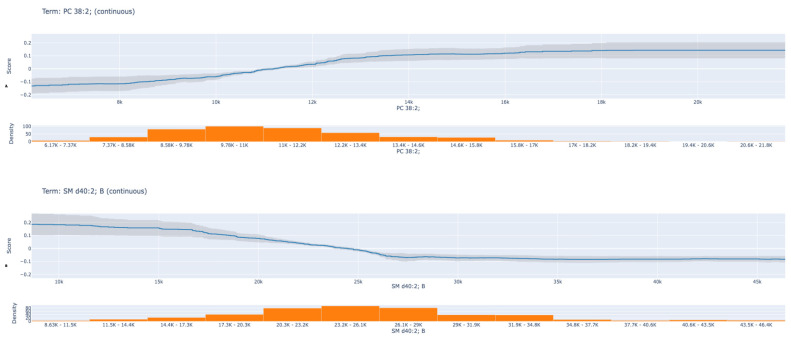
Partial dependency plots (PDPs) of the two most influential lipid biomarkers identified by the EBM model. (**A**) Phosphatidylcholine (PC 38:2) and (**B**) Sphingomyelin (SM d40:2 B) demonstrate opposite concentration-dependent effects on predicted HCC probability. The *x*-axis represents lipid concentration (arbitrary units, a.u.) derived from LC-QTOF-MS analysis. The *y*-axis indicates the EBM model’s predicted contribution to HCC classification probability. Higher PC (38:2) levels increase the likelihood of a positive HCC prediction, while higher SM d40:2 B concentrations show an inverse relationship. Abbreviations: PC—phosphatidylcholine; SM—sphingomyelin.

**Figure 3:**
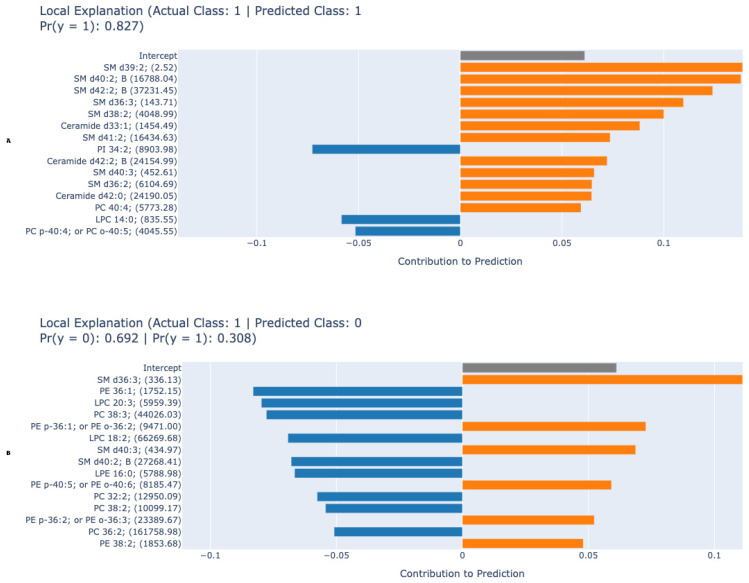
Local explanation plots for individual predictions. (**A**) Correct positive prediction showing lipid contributions. (**B**) False negative prediction demonstrating how specific lipid levels reduced the HCC probability. Bars represent individual lipid contributions (positive or negative) to the model prediction probability. Blue bars indicate lipids contributing to HCC prediction, while orange bars denote those opposing the prediction. Key biomarkers such as PC (38:2), SM d40:2 B, and LPC (18:2) are visually emphasized. Abbreviations: PC—phosphatidylcholine; SM—sphingomyelin; LPC—lysophosphatidylcholine; Cer—ceramide; PE: Phosphatidylethanolamine.

Figure 2 shows the two most important lipids in the EBM global explanations, PC 38:2 and SM d40:2, in the EBM partial dependency plots. Figure 3A shows that the PC 38:2 lipid contributes slightly negatively to the model score at low concentration ranges, but as the concentration increases, the score shifts toward the positive direction. This suggests that higher PC 38:2 levels increase the model’s probability of predicting a positive class. In contrast, for the SM d40:2 B lipid, a high score contribution was observed at low concentrations, but as concentration increased, the contribution gradually decreased and showed a slight negative effect at high levels. This pattern suggests that PC 38:2 and SM d40:2 B lipids may have different biological roles and may exhibit opposing biomarker effects in liver cancer classification.

Figure 3A,B show local EBM explanations for one example each that the model correctly predicted as positive and incorrectly predicted as negative. Local explanation graphs show more clearly the contribution of these lipids to the prediction in individual examples. In the first example (A), where the model made a correct positive prediction, SM d39:2, SM d40:2 B, SM d42:2 B, while phosphatidylinositol and phosphatidylcholine derivatives such as PI 34:2 and PC p-40:4 contributed negatively to the prediction result. In the second example (B), in cases where the model made a false negative prediction, the patient’s lipid levels such as SM d36:3, and PC 38:3 contributed negatively, reducing the probability of a positive class, while some phosphatidylethanolamine types and sphingomyelins contributed positively. These findings demonstrate that the EBM model considers the different effects of specific lipid subclasses when determining classification decisions at both the global and local scales, thereby strengthening the model’s biological interpretability.

## 4. Discussion

The integration of lipidomics with explainable ML models in this study has yielded robust insights into the metabolic dysregulation associated with HCC. Our results demonstrate that the EBM algorithm outperformed other ML models (XGBoost, Random Forest, CatBoost, SVM) in classifying HCC with 87.0% accuracy, 87.1% F1-score, 87.7% sensitivity, 86.3% specificity, and an AUC of 0.918. The superior performance of EBM over established “black-box” models like XGBoost underscores the critical importance of transparency in clinical decision-support tools, where understanding the “why” behind a prediction is as crucial as the prediction itself.

### 4.1. Machine Learning Model Performance and Interpretability

EBM’s dominance over other ML models (Table 2) can be attributed to its additive modeling structure, which quantifies feature contributions while retaining high accuracy. Unlike “black-box” algorithms (e.g., XGBoost, AUC: 0.866), EBM leverages pairwise interactions to generate global and local explanations without sacrificing performance. For instance, global explanations (Figure 1) ranked PC (38:2), SM d40:2 B, PC (40:4), LPC (18:2), PC (36:2), and PC (36:1) as top contributors, respectively. This synergy between statistical significance and ML-driven feature importance enhances biological plausibility, as EBM highlights lipids independently validated via traditional methods. The partial dependency plots (Figure 2) further revealed nuanced biomarker behavior: PC (38:2) concentrations positively correlated with HCC risk, while SM d40:2 B exhibited an inverse relationship. Such patterns suggest divergent pathophysiological roles—PCs may promote membrane remodeling in proliferating tumor cells, whereas SMs could reflect disrupted sphingolipid metabolism in advanced liver disease [9,11,21]. The consistent outperformance of the EBM across all metrics (Accuracy, F1-Score, Sensitivity, Specificity, and AUC) is a key finding. While ensemble methods like XGBoost and Random Forest are renowned for their predictive power, their complex, non-linear interactions often render them inscrutable. The EBM circumvents this limitation through its Generalized Additive Model (GAM) framework, which combines the performance of modern boosting techniques with the interpretability of linear models. Our results confirm that high accuracy need not be sacrificed for explainability. The global and local explanations provided by the EBM (Figure 1, Figure 2 and Figure 3) offer an unprecedented level of insight, allowing clinicians and researchers to see which specific lipids drive each classification decision. This transparency is essential for building the trust required for clinical adoption and for generating actionable biological hypotheses [29].

### 4.2. Lipid Biomarkers: Pathobiological Implications

The lipid species highlighted by the EBM are not merely statistical artifacts; they are central players in the metabolic reprogramming that characterizes HCC. Our findings point to profound disruptions in two key lipidomic domains: phospholipid remodeling and sphingolipid metabolism.

Phosphatidylcholines (PCs) and Lysophosphatidylcholines (LPCs): The prominence of PCs such as PC (38:2) and PC (36:2) aligns with their known role as major structural components of cell membranes. In rapidly proliferating cancer cells, the demand for membrane building blocks is immense, leading to altered PC metabolism. Elevated levels of certain PCs may reflect enhanced de novo synthesis pathways to support unchecked cell division and membrane biogenesis. Furthermore, PCs are reservoirs for signaling molecules. The conversion of PCs to LPCs by phospholipase A2 enzymes generates lipid mediators that influence a wide range of processes. The significant reduction in LPC (18:2) observed in our study is particularly noteworthy. LPCs, especially those containing polyunsaturated fatty acids like linoleic acid (18:2), have been shown to possess immunomodulatory properties. Lower levels of LPC (18:2) have been correlated with an immunosuppressive tumor microenvironment, characterized by impaired T-cell function and poorer prognosis in HCC. This case suggests that our model may be capturing not just tumor-centric metabolic shifts, but also the associated immune dysregulation [30,31].

Sphingolipid Metabolism as a Core Dysregulated Pathway: The most compelling biological insights from our analysis revolve around sphingolipids. The EBM identified specific sphingomyelins (SM d40:2 B, SM d42:2 B) and ceramides (Cer d42:2 B) as top features. Sphingolipids function within a critical “rheostat” system where the balance between pro-proliferative sphingosine-1-phosphate (S1P) and pro-apoptotic ceramides determines cell fate [32,33].

The depletion of specific sphingomyelins in HCC patients could indicate their accelerated hydrolysis by sphingomyelinases to generate ceramides. This is a well-documented cellular response to various stresses, including metabolic stress and inflammatory signaling, which are hallmarks of chronic liver disease and hepatocarcinogenesis. The generated ceramides can then promote apoptosis, senescence, or inflammation. However, cancer cells often develop mechanisms to evade ceramide-induced cell death, for instance, by converting ceramides to S1P or glycosylceramides. The specific ceramide species highlighted by our model (e.g., Cer d42:2 B) may represent particular isoforms that are differentially regulated in HCC, potentially offering new mechanistic clues [34]. This disruption of the sphingolipid rheostat is increasingly recognized as a pivotal event in the progression from NAFLD to HCC. In the context of NAFLD, chronic metabolic overload and oxidative stress in hepatocytes can activate sphingomyelinases, leading to ceramide accumulation. This contributes to insulin resistance, hepatocyte apoptosis, and the recruitment of inflammatory cells, driving fibrosis and ultimately creating a pro-carcinogenic microenvironment. Our findings, derived from blood-based lipidomics, likely reflect this systemic and hepatic sphingolipid dysregulation, offering a non-invasive window into these core pathophysiological processes [35,36,37].

The pronounced alterations in sphingolipids, particularly the reduction in SM d40:2; B and the significant role of ceramides, point to a fundamental disruption in the ceramide-sphingomyelin balance that is critical for liver homeostasis. Sphingomyelins are membrane components that can be hydrolyzed to generate ceramides, potent pro-apoptotic and anti-proliferative signaling molecules. The observed depletion of specific sphingomyelins in HCC may reflect either increased consumption for ceramide production or impaired synthesis, both of which would alter membrane fluidity and signal transduction. Concurrently, the model’s emphasis on ceramide species (e.g., Cer d42:2 B) suggests a compensatory or stress-induced activation of ceramide-generation pathways—such as through acid sphingomyelinase—which can drive hepatocyte apoptosis, promote inflammation, and create a tumor-permissive microenvironment. This sphingolipid rheostat imbalance is increasingly recognized as a key feature in NAFLD-to-HCC progression, where chronic metabolic stress disrupts lipid-mediated signaling, favoring survival of damaged hepatocytes and ultimately carcinogenesis. Recent research highlights that dysregulated sphingolipid metabolism, particularly an imbalance in the sphingolipid rheostat (the ratio of ceramide to sphingosine-1-phosphate, S1P), is a key feature in the progression from NAFLD to HCC. Lipidomic analyses also reveal shifts in sphingomyelin metabolism during carcinogenesis, further supporting the role of sphingolipid dysregulation in disease advancement 3. These findings suggest that targeting sphingolipid metabolism could offer new diagnostic or therapeutic strategies for NAFLD-related HCC [38,39].

### 4.3. Clinical Translations

The transition from biomarker discovery to clinical utility hinges on interpretability and validation. The EBM framework excels in this regard. The partial dependency plots (Figure 2) move beyond simple “up/down” regulation, revealing nuanced, concentration-dependent relationships between lipid levels and HCC risk. The positive correlation of PC (38:2) and the inverse relationship of SM d40:2 B with model score suggest their opposing roles, which could be crucial for understanding patient-specific disease drivers.

Furthermore, the local explanations (Figure 3) are a gateway to personalized medicine. In a clinical setting, a physician could, in theory, review a local explanation for a patient’s positive test result to understand which specific lipid abnormalities were most influential. Conversely, in a false-negative case, the local explanation could reveal a conflicting lipid signature—for example, a strong negative contribution from a specific PC outweighing a positive contribution from a ceramide—thereby highlighting biological heterogeneity and potentially identifying a patient subgroup with a distinct metabolic phenotype. This level of insight is impossible to obtain from a black-box model and represents a significant step towards personalized diagnostic profiling.

### 4.4. Limitations and Future Directions

First, the dataset lacked granular clinical metadata in the model (e.g., etiology of liver disease, tumor stage), limiting subgroup analyses. Future studies should validate these lipids in etiologically diverse cohorts (e.g., HBV- vs. NAFLD-related HCC). Second, while LC-QTOF-MS enabled broad lipid coverage, absolute quantification was not performed; semi-quantitative data may obscure concentration-dependent biomarker thresholds. EBM’s reliance on additive models could underrepresent complex interactions among lipids. Integration with deep learning architectures may resolve this issue [20,39]. Furthermore, the semi-quantitative nature of the lipidomic data, expressed as integrated peak areas in arbitrary units, should be emphasized. While this approach is highly effective for discovering relative differences between groups, it does not provide absolute concentrations. This inherently limits the ability to define universal clinical cut-off values based on our current results. Future validation studies employing targeted absolute quantification will be essential to establish such clinically actionable thresholds. A further limitation is that the model was developed and evaluated on a single dataset. To truly assess its clinical utility and generalizability, external or clinical validation on an independent, multi-center cohort with varying demographics and etiologies of liver disease is essential.

## 5. Conclusions

This study successfully establishes a transparent and accurate framework for HCC diagnosis by integrating lipidomics with an EBM. We have identified a panel of lipid biomarkers—notably PCs, LPCs, and key sphingolipids—that reflect core pathomechanisms of HCC, including unchecked membrane synthesis, immune evasion, and a disrupted sphingolipid rheostat. The EBM model provides both global and local interpretability, offering a level of insight that bridges the gap between computational prediction and biological understanding. Future work must focus on the external validation of this lipid signature in etiologically diverse cohorts, its absolute quantification, and mechanistic studies to confirm its role in hepatocarcinogenesis. This approach holds immense promise for developing trustworthy, non-invasive liquid biopsies that can improve the early detection of HCC and ultimately patient outcomes. Future work should prioritize multi-center validation on independent datasets to confirm the robustness and transportability of this lipid biomarker panel and the EBM framework before clinical implementation can be considered.

## Figures and Tables

**Table 1:** Statistics on demographic and clinical features.

Variable	HCC	Control
Age (years), mean ± SD	57.8 ± 5.6	56.9 ± 5.2
AGE DISTRIBUTION		
≤54 years, n (%)	61 (27.9)	65 (29.7)
55–59 years, n (%)	90 (41.1)	99 (45.2)
60–64 years, n (%)	48 (21.9)	42 (19.1)
≥65 years, n (%)	20 (9.1)	13 (5.9)
Body Mass Index (kg/m^2^), mean ± SD	27.2 ± 4.1	25.8 ± 3.6
BMI CATEGORIES		
<25 kg/m^2^, n (%)	61 (27.8)	84 (38.3)
25 to <30 kg/m^2^, n (%)	108 (49.3)	106 (48.4)
≥30 kg/m^2^, n (%)	50 (22.8)	29 (13.2)
EDUCATIONAL LEVEL		
Elementary/no vocational, n (%)	45 (20.5)	69 (31.5)
Elementary/vocational, n (%)	112 (51.1)	104 (47.9)
Beyond elementary, n (%)	62 (28.3)	45 (20.5)
SMOKING INTENSITY CATEGORIES		
<25 pack-years, n (%)	52 (23.7)	70 (31.9)
25–34 pack-years, n (%)	44 (20.0)	51 (23.2)
35–44 pack-years, n (%)	49 (22.3)	52 (23.7)
≥45 pack-years, n (%)	74 (33.7)	46 (21.0)
ALCOHOL CONSUMPTION CATEGORIES		
Non-drinker, n (%)	14 (6.3)	14 (6.3)
>0 to <1 drink/day, n (%)	68 (31.0)	105 (47.9)
1 to <2 drinks/day, n (%)	60 (27.4)	54 (24.7)
≥2 drinks/day, n (%)	62 (28.3)	35 (16.4)
DIABETES STATUS		
No diabetes, n (%)	198 (90.4)	214 (97.7)
Diabetes present, n (%)	21 (9.6)	5 (2.3)

Values are presented as mean ± standard deviation (SD) for continuous variables or frequency (percentage) for categorical variables.

**Table 2:** Comparative performance analysis results of machine learning models.

Metrics/Model	SVM	CatBoost	Random Forest	XGBoost	EBM
Accuracy	0.765 (0.725–0.805)	0.785 (0.747–0.824)	0.797 (0.759–0.834)	0.806 (0.769–0.843)	0.870 (0.838–0.901)
F1-Score	0.775 (0.735–0.814)	0.793 (0.755–0.831)	0.804 (0.766–0.841)	0.812 (0.776–0.849)	0.871 (0.839–0.902)
Sensitivity	0.808 (0.75–0.858)	0.822 (0.765–0.87)	0.831 (0.775–0.878)	0.840 (0.785–0.886)	0.877 (0.826–0.917)
Specificity	0.721 (0.657–0.78)	0.749 (0.686–0.805)	0.763 (0.701–0.817)	0.772 (0.71–0.826)	0.863 (0.81–0.906)
AUC	0.828 (0.764–0.893)	0.848 (0.785–0.910)	0.858 (0.797–0.919)	0.866 (0.806–0.927)	0.918 (0.870–0.966)

## Data Availability

The raw data supporting the conclusions of this article will be made available by the authors on request.

## Supplementary Materials

The following supporting information can be downloaded at: https://www.mdpi.com/article/10.3390/metabo15110716/s1, Table S1. The fold change analysis of semi-quantitative lipid measurements in hepatocyte carcinoma patients and matched controls.; Figure S1. Volcano plot showing the distribution of lipid species based on log2 fold change (x-axis) and -log10 adjusted *p*-values (y-axis) between hepatocellular carcinoma patients and matched controls; Figure S2. PLS-DA score plot demonstrating the separation between hepatocellular carcinoma patients and control samples based on lipidomic profiles; Figure S3. Cross-validation performance metrics for the PLS-DA model across different numbers of components; Figure S4. PLS-DA model permutation test result; Figure S5. VIP scores from PLS-DA analysis showing the top contributing lipid species for group discrimination; Figure S6. Heatmap displaying the hierarchical clustering of differentially expressed lipid species across hepatocellular carcinoma patients and matched controls, with color intensity representing normalized abundance levels.
