# Supplementary material for: Identification of a Novel Lipidomic Biomarker for Hepatocyte Carcinoma Diagnosis: Advanced Boosting Machine Learning Techniques Integrated with Explainable Artificial Intelligence
Source: Metabolites. 2025 Nov 1;15(11):716. doi: 10.3390/metabo15110716 (PMC12654175; doi:10.3390/metabo15110716)
Supplement: Supplementary file 1 [file metabolites-15-00716-s001.zip › metabolites-3937353-supplementary.pdf]

Supplemantary File

Table S1. The fold change analysis of semi-quantitative lipid measurements in hepatocyte carcinoma patients and matched controls.

Name	log_2FC	adj. p value	$log_{10}p$ value	Regulation
SM d39:2	-0.434	<0.001	11.325	Down
SM d40:2 B	-0.285	<0.001	11.325	Down
PC 40:4	0.314	<0.001	97.888	Up
PC 32:1	0.471	<0.001	88.234	Up
SM d36:3	-0.346	<0.001	80.664	Down
LPC 18:2	-0.274	<0.001	79.928	Down
SM d37:1	-0.272	<0.001	79.468	Down
FA 16:1 (palmitoleic acid)	0.431	<0.001	52.499	Up
PE 34:1	0.333	<0.001	48.685	Up
PC 40:5 B	0.286	<0.001	48.291	Up
Ceramide d42:0	0.275	<0.001	43.675	Up
FA 22:2 (docosadienoic acid)	0.599	<0.001	41.209	Up
Ceramide d40:0	0.348	<0.001	2.785	Up
PC p-42:5; or PC o-42:6	-0.297	0.003	2.513	Down
SM d42:0	0.280	0.008	20.636	Up

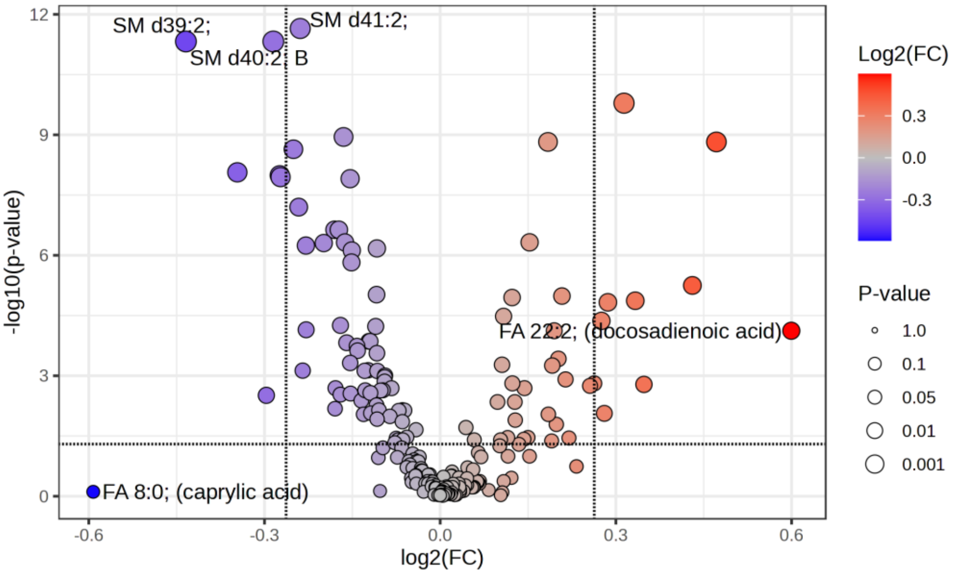

Figure S1. Volcano plot showing the distribution of lipid species based on $\log_2$ fold change (x-axis) and $-\log_{10}$ adjusted p-values (y-axis) between hepatocellular carcinoma patients and matched controls.

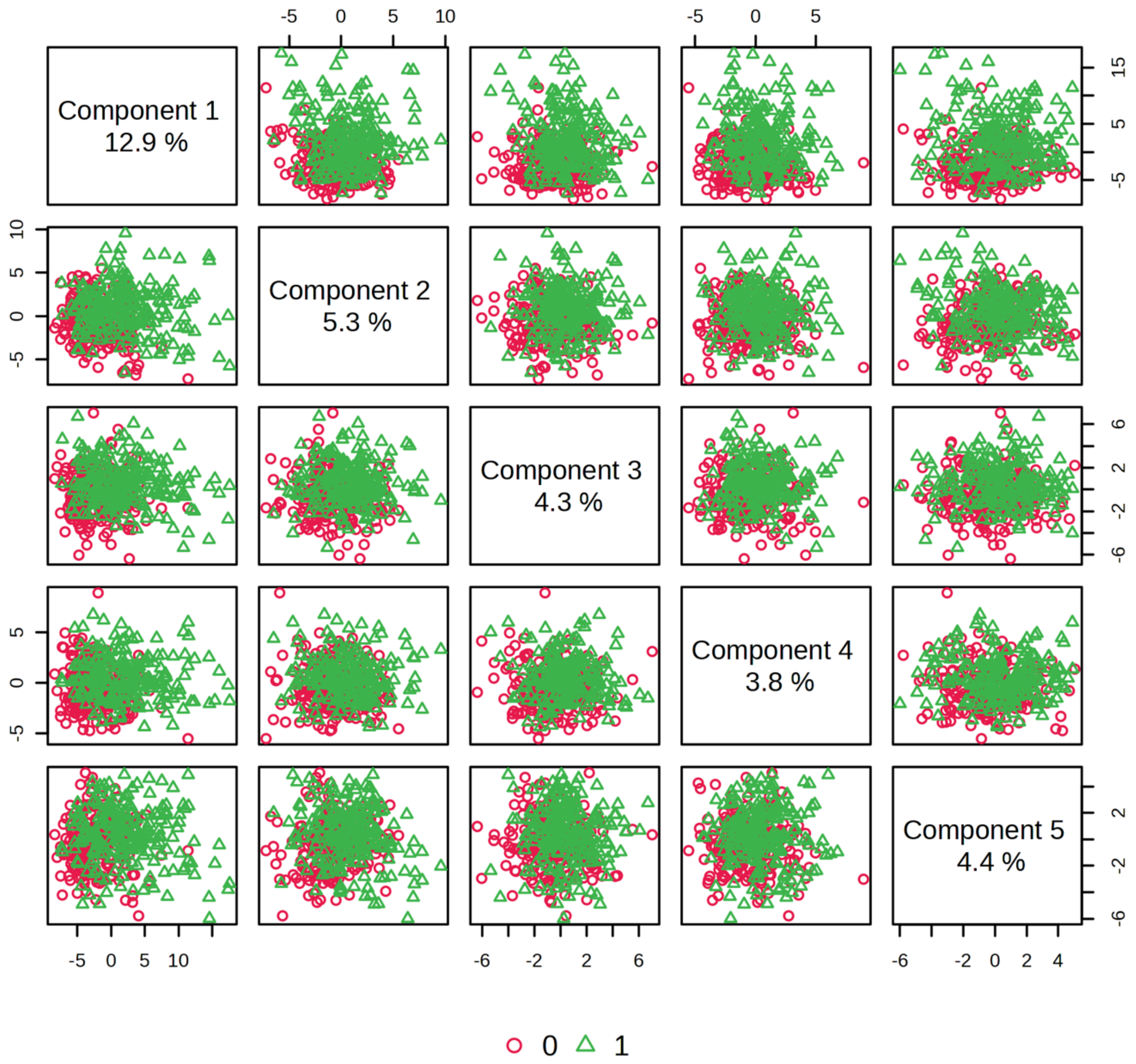

Figure S2. PLS-DA score plot demonstrating the separation between hepatocellular carcinoma patients and control samples based on lipidomic profiles.

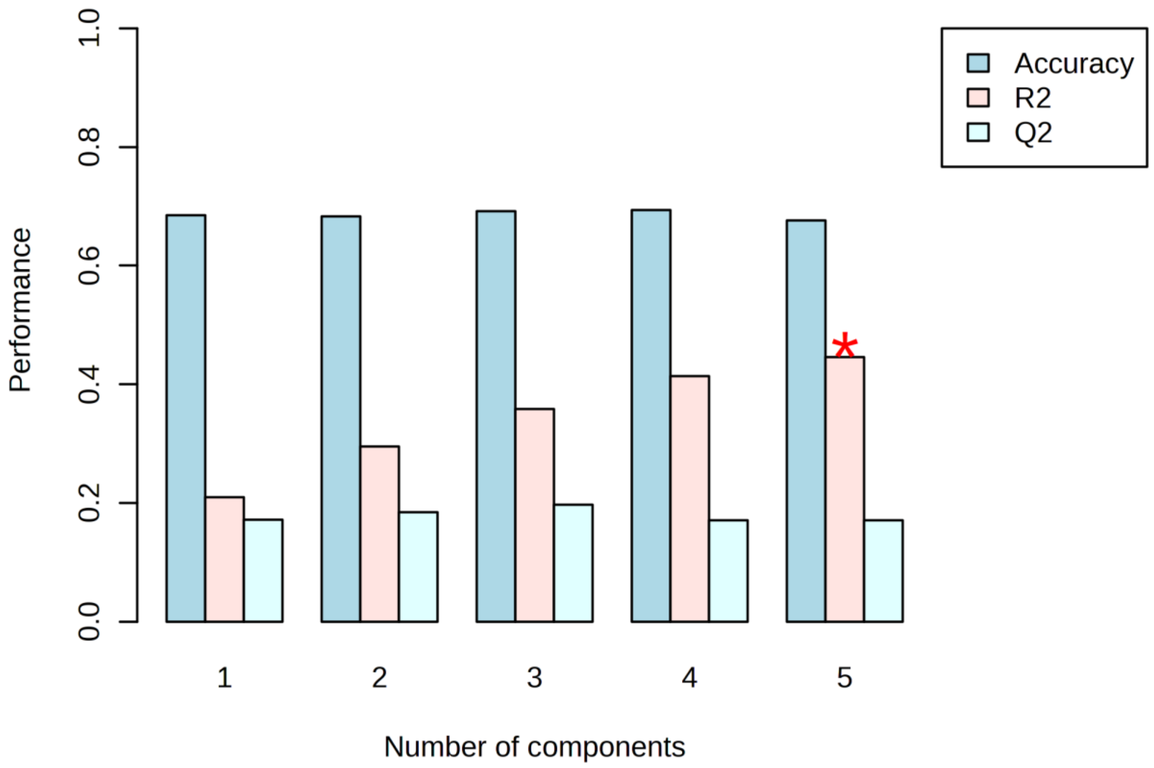

Figure S3. Cross-validation performance metrics for the PLS-DA model across different numbers of components.

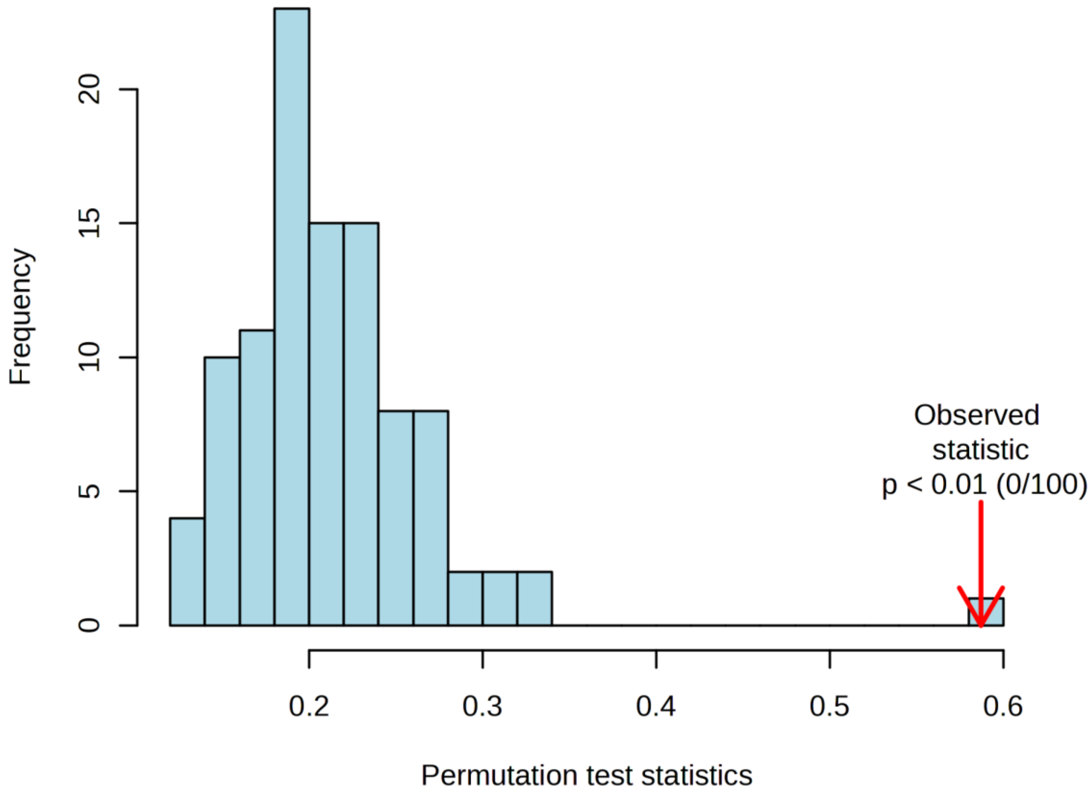

Figure S4. PLS-DA model permutation test result

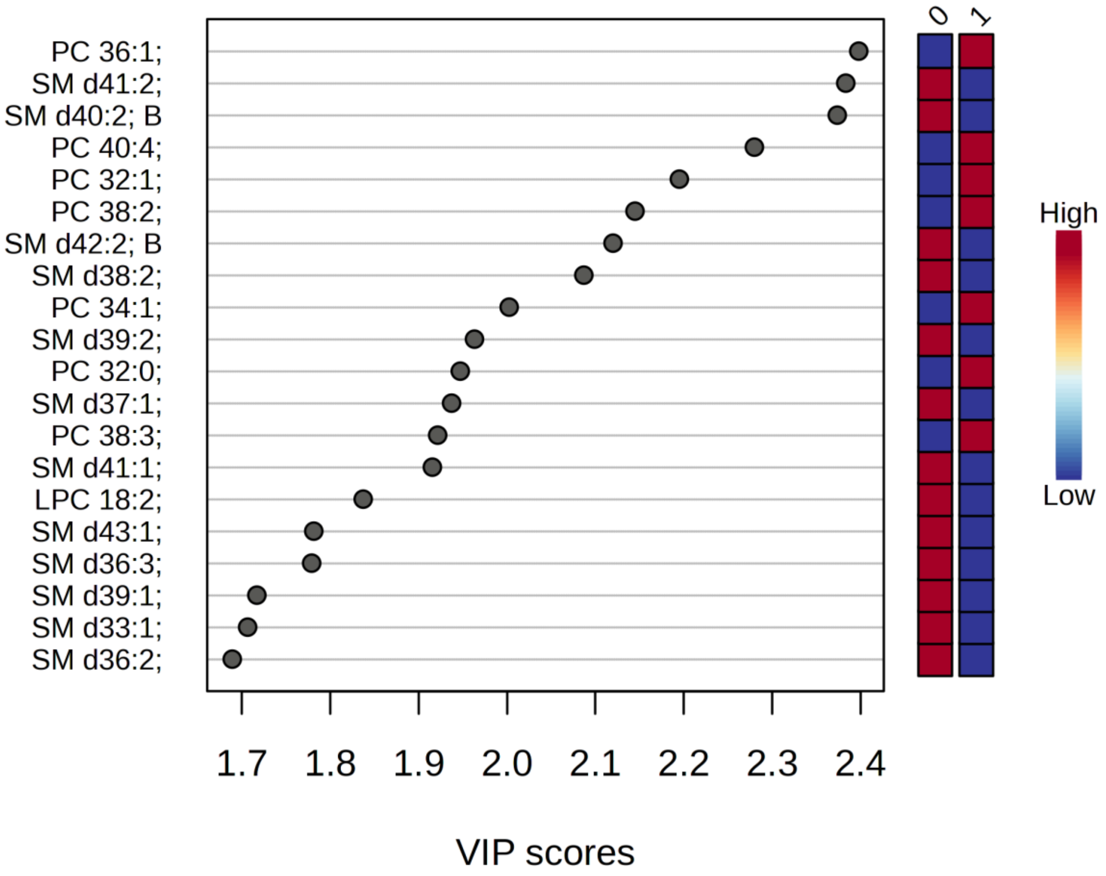

Figure S5. VIP scores from PLS-DA analysis showing the top contributing lipid species for group discrimination.

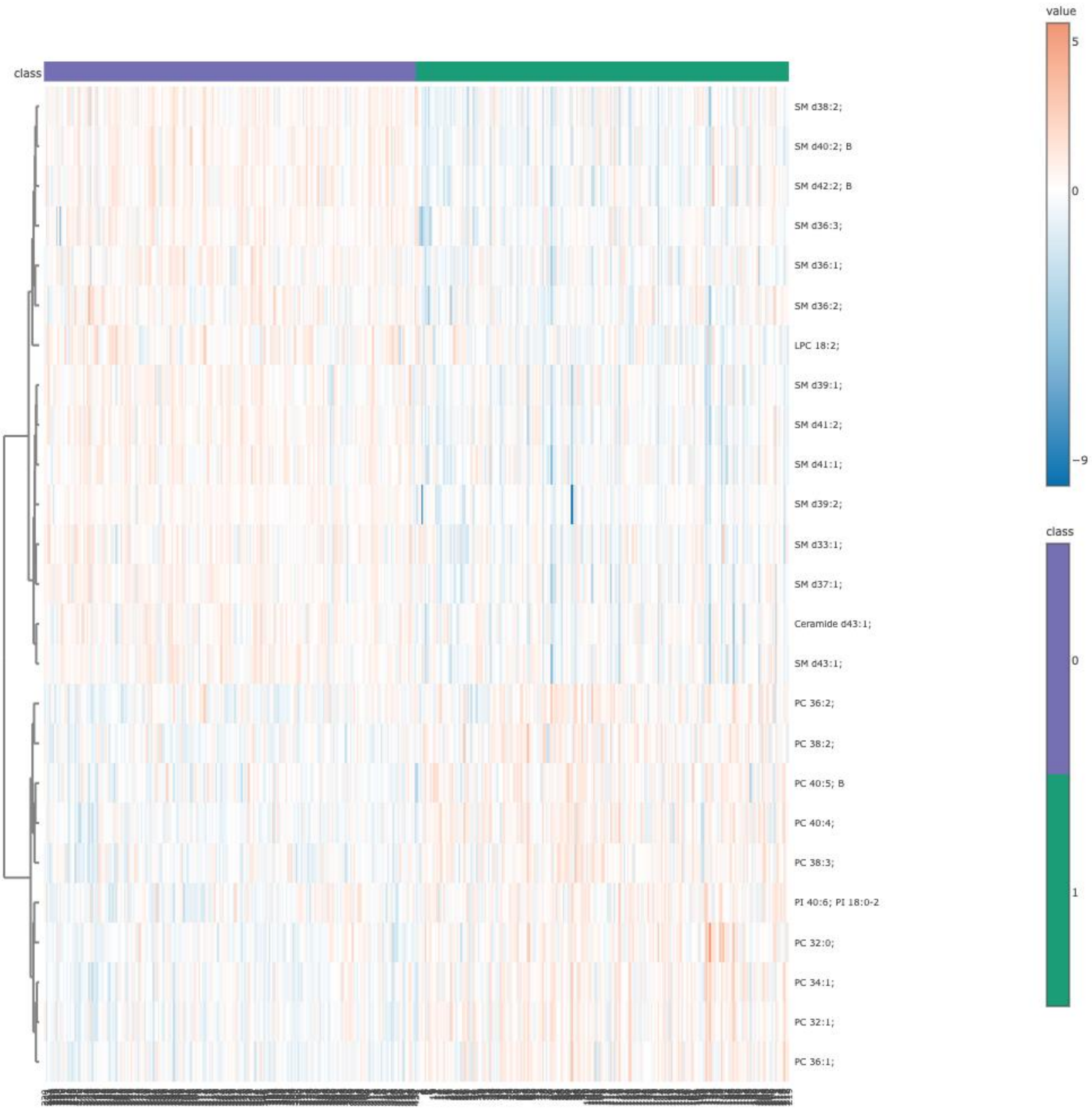

Figure S6. Heatmap displaying the hierarchical clustering of differentially expressed lipid species across hepatocellular carcinoma patients and matched controls, with color intensity representing normalized abundance levels.
